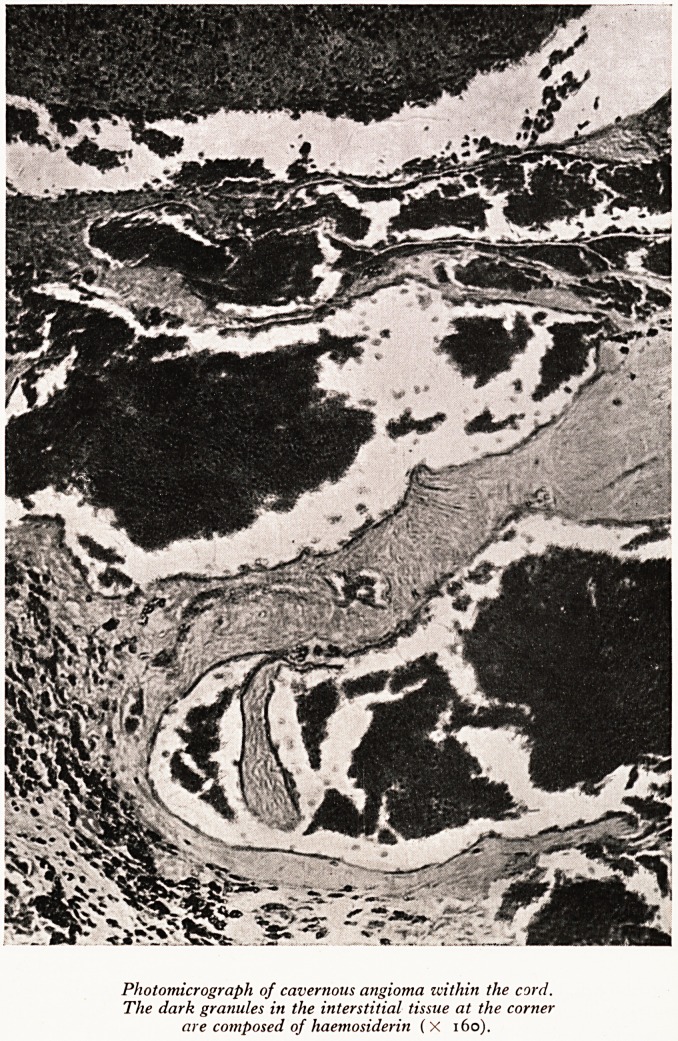# Haemorrhage from a Cavernous Haemangioma of the Cord Clinically Resembling an Ascending Myelitis

**Published:** 1955-11

**Authors:** T. F. Hewer


### HAEMORRHAGE FROM A CAVERNOUS HAEMANGIOMA OF THE CORD CLINICALLY RESEMBLING AN ASCENDING MYELITIS

*A Clinical Pathological Conference of the University of Bristol Medical School*

CHAIRMAN: PROFESSOR T. F. HEWER

*Professor T. F. Hewer:* The patient was a man of 68. Dr. A. G. Morris has sent me a letter in which he says that he knew the man for a good many years and had only attended him for mild respiratory infections until the last occasion when he had acute retention, so he sent him into hospital immediately.

*Mr. R. V. Cooke:* This man was admitted under my care because of retention of urine for two days; the House Surgeon found that there were neurological signs. A physician then saw him and treated him in one of my beds. I did nothing for him and the whole course of events was under medical guidance.

*Dr. J. E. Cates:* As you have heard, this man came in to hospital on the 24th March as an emergency with complete retention of urine for two days before admission. He gave the story that he was quite well until four weeks before admission when he noticed that he had a tendency to trip over things and that he could not keep his feet clear of the ground when walking. Then he noticed that his left leg felt numb in bed at night. About three weeks before admission he had a tight feeling around his tummy. He then lost sensation below the level of the constriction. On examination, there was a loss of power below the level of the waist with, at first, some spasticity and, to begin with, exaggerated knee and ankle jerks and, later, absent reflexes. All sensation was lost below the waist, but there was no band of hyperaesthesia. About two days later the sensory loss climbed up his body and he could not feel anything below the level of his shoulders. At that stage we were a bit out of our depth so we asked Dr. Campbell to see him.

What could be the disorder of function causing these physical signs? There was involvement both of sensory pathways with complete anaesthesia, and of the motor tracts because of the spasticity and loss of power. I presumed that the fact that he later developed flaccidity and hypotonia indicated that other tracts were affected as well.

Where was the lesion? There seemed to be no doubt from the history and from physical signs that the disorder of function had climbed up the spinal cord. His subsequent course and results of investigations were those that you would expect in a case paralysed from the neck down: he developed respiratory infection with high temperature and purulent sputum; he developed a large bedsore. He also had a urinary infection which presumably spread from his bladder into his kidneys and was accompanied by rigors. He progressively went downhill, lost weight and fortunately died about five weeks after admission, on the 6th May.

*Dr. A. M. G. Campbell:* I am sure I have very little to add to what Dr. Cates has already told you. We thought that he probably had myelitis. At that particular time Dr. Hoffman was preparing a paper on myelitis, so we asked him to see the case and I would like him to talk about what he saw at the time.

*Dr. H. L. Hoffman:* Three or four years ago I saw a similar clinical picture in a young woman at one of these meetings, and it is because I am particularly interested in these cases that Dr. Campbell asked me to see this one. As you will hear later, this is a very good lesson not to diagnose everything as the particular condition you happen to be interested in at the particular moment! I thought this man was a case of acute necrotic myelopathy and that the fact that he had no knee and ankle jerks on one side and diminished ankle jerks on the other and one absent plantar response indicated that there was no function in the lower part of the spinal cord because there was no surviving cord tissue to produce them. This is quite distinct from transverse myelitis where the cord carries on a separate function below the lesion. That is what I thought he was at that particular time and he fitted in exactly with the other five cases I have seen.

*Mr. R. V. Cooke:* I saw this case a few days after admission and did suggest that it might be a spinal tumour.

*Dr. Campbell:* This was unlikely in view of complete absence of C.S.F. changes; there was no block or rise in proteins. There was also the altering level which is uncharacteristic in a tumour, although it does occur sometimes. We had no evidence here that there was a tumour. The other point was that he had lost both knee and ankle jerks.

*Professor Hewer* then presented the post-mortem findings:

This man was extremely thin and looked as if he had been ill for much longer than he had been. There was an enormous sacral bedsore, also a bedsore on the left hip, left knee and left heel—there were none on the right side. The only changes other than those in the C.N.S. were a fairly large pulmonary embolism in the left pulmonary artery which had come from the left femoral vein which, with the popliteal vein, was entirely thrombosed. This was associated with phlebitis. Other changes included a recent ascending infection of both ureters and an acute cystitis. There was no question of any tumours anywhere within the abdominal or thoracic cavities. The skull and brain showed no change. When I came to the spine I was fully expecting to find an acute necrotizing myelitis. I removed the laminae of the spine noting there was no tumour in any of them, paying special attention to T.2–3. There was no sign of any tumour. On examining the theca, there was no sign of any swelling: it was quite loose and there was no block. I then opened up the theca and found that, at the level of T.2, the posterior aspect of the spinal cord itself was bulging and measured 18 mm. in diameter at T.2, 16 mm. at T.1 and 13 mm. at T.3. This was a considerable amount of swelling but there was no discoloration and no sign of exudate. The texture of the cord below that point felt quite firm: that was against the diagnosis of ascending necrotizing myelitis, in which case the whole cord would have been very tense. There was not complete necrosis of the cord, so something had happened inside the cord substance at that level. As it might be a vascular accident I carefully examined the vessels on the posterior aspect of the cord, before removing it, and found thay were all patent: blood could be moved up and down them with the tip of a finger. On removing the cord from the spinal canal one could see a discoloured area anteriorly which was evidently due to haemorrhage within the cord (Plate XXII). On this anterior aspect the superficial vessels were also patent. After fixation I cut transverse sections through the cord at intervals and found a considerable fresh haemorrhage in the cord substance, maximal at T.2. Histologically a section at this level showed a large cavernous haemangioma occupying the central area of the T.2 segment (Plate XXIII). Haemorrhage from this angioma had occurred in the past and haemosiderin deposits were seen alongside the vessels. The main haemorrhage was recent and was confined to the cord substance—a condition of haematomyelia.

These cavernous haemangiomata in the central nervous system not infrequently give rise to haemorrhage. Judging by the presence of all the haemosiderin in the section there had been small haemorrhages in the cord before the final fatal one. This would explain the onset of symptoms some two and a half months before death.

*Mr. Cooke:* Could we have done anything? How far down the cord had the haemangioma spread?

*Professor Hewer:* It was a little difficult to determine because of the haemorrhage, but the haemangioma occupied a large part of T.2 and extended a little way into both T.1 and T.3. It was difficult to see the limits of the angioma: there was haemorrhage as far as the lower level of T.4 but no angioma. The angioma could not possibly have been removed without causing a paraplegia.

*Question:* Do you think Dr. Hoffman could explain the abolished knee and ankle jerks?

### PLATE XXII

*Spinal cord and theca: anterior view showing haemorrhage at level of second thoracic segment.*

PLATE XXIII

*Photomicrograph of cavernous angioma within the cord. The dark granules in the interstitial tissue at the corner are composed of haemosiderin* (× 160).

*Dr. Hoffman:* I saw the man after twelve days and it is conceivable that if the final haemorrhage was a fairly acute affair, which I think it was, spinal shock would be the explanation. The bleeding at first is under extremely low pressure and that was probably responsible for his symptoms in the early days. Then he had several sudden episodes which produced the condition of spinal shock. In this condition, below the level of the lesion, everything is abolished, and in about three weeks time the transverse lesion manifests itself and the jerks come back. The other thing which is difficult to explain is why the central lesion of the cord should produce an ascending sensory loss, which is exactly the reverse of what you would expect. The main part of the tumour itself was fairly central but the haemorrhage occurred towards the left lateral side of the cord, involving the outer part of the spinothalamic tract and so the sensory loss spread upwards: this is the only explanation I can give.

*Question:* Was the spinal cord well below the level of the lesion perfectly normal? Professor Hewer said that it was.

*Dr. Hoffman:* I noticed on examination that the muscles of his hands were wasted. It is conceivable that some wasting might have occurred before the haemorrhage. The upper level of T.1 is the site of the nerve roots supplying the small hand muscles, so it is possible that there was just some evidence, if one had been quick enough to realise that there was an underlying tumour: Nothing could have been done.

*Question:* If you are born with an angioma anywhere, does it grow during life?

*Dr. Campbell:* I have had one case recently in which five or six angiomata, including one on the skin, suddenly began to grow.

*Mr. Cooke:* Were they present from birth?

*Dr. Campbell:* Yes.

*Professor Hewer:* There are a great many varieties of angiomatous malformations but they are practically all congenital in origin and they grow with the growth of the body. Subsequent increase in size is often due to haemorrhage within them.

*Question:* Were there no other vascular changes in this case?

*Professor Hewer:* No, none at all.

The conclusion was that this was an example of a congenital vascular malformation—a hamartoma—of the spinal cord and that bleeding took place gradually over a period of some three months, causing a gradual onset of signs and symptoms of a cord lesion. Eventually a massive haemorrhage caused a haematomyelia but as it did not rupture into the subarachnoid space there was no sign in the cerebrospinal fluid.

## Figures and Tables

**Figure f1:**
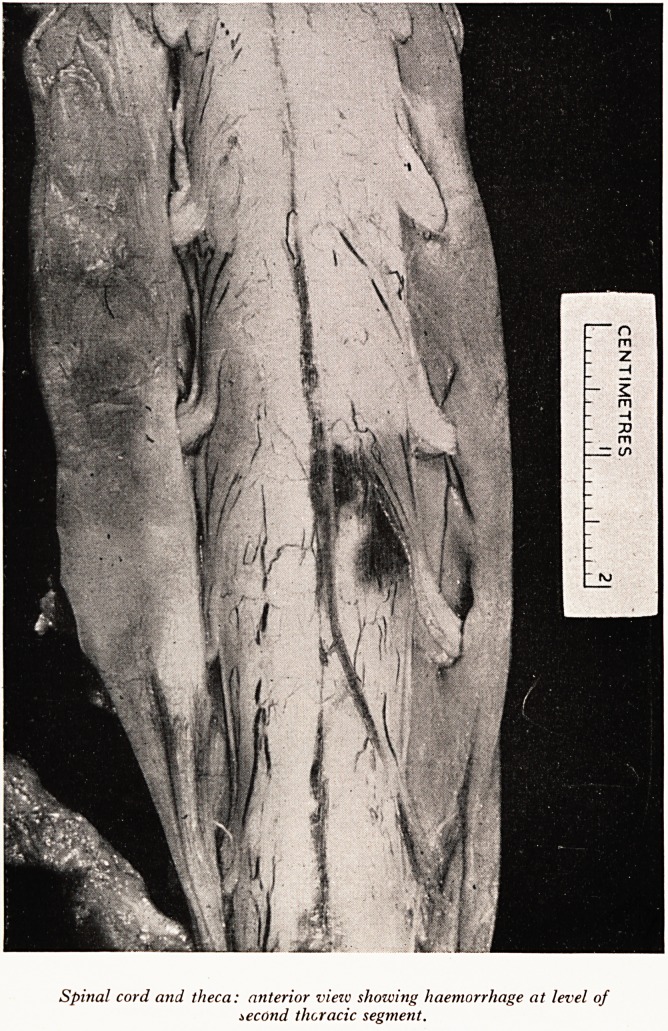


**Figure f2:**